# Abdominal Wall Defects—Current Treatments

**DOI:** 10.3390/children8020170

**Published:** 2021-02-23

**Authors:** Isabella N. Bielicki, Stig Somme, Giovanni Frongia, Stefan G. Holland-Cunz, Raphael N. Vuille-dit-Bille

**Affiliations:** 1 Department of Pediatric Surgery, University Children’s Hospital of Basel (UKBB), 4056 Basel, Switzerland; Isabella.bielicki@ukbb.ch (I.N.B.); Stefan.holland-cunz@ukbb.ch (S.G.H.-C.); 2 Department of Pediatric Surgery, University Children’s Hospital of Colorado, Aurora, CO 80045, USA; stig.somme@childrenscolorado.org; 3 Section of Pediatric Surgery, Department of General, Visceral and Transplantation Surgery, 69120 Heidelberg, Germany; giovanni_frongia@gmx.de

**Keywords:** abdominal wall defect, gastroschisis, omphalocele, treatment

## Abstract

Gastroschisis and omphalocele reflect the two most common abdominal wall defects in newborns. First postnatal care consists of defect coverage, avoidance of fluid and heat loss, fluid administration and gastric decompression. Definitive treatment is achieved by defect reduction and abdominal wall closure. Different techniques and timings are used depending on type and size of defect, the abdominal domain and comorbidities of the child. The present review aims to provide an overview of current treatments.

## 1. Gastroschisis

### 1.1. Introduction

**Figure 1:**
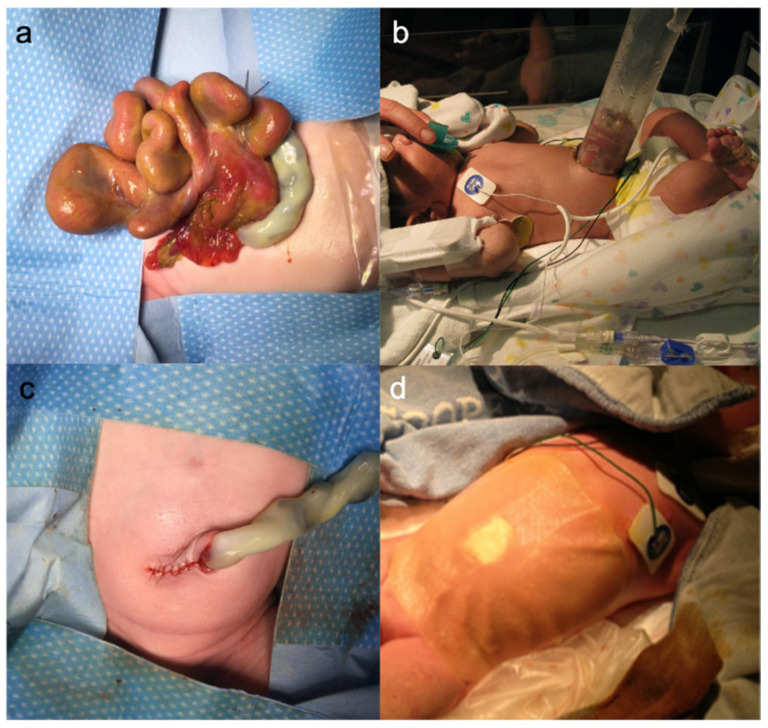
Gastroschisis: (**a**) Herniated intestine and greater omentum; (**b**) Silo treatment; (**c**) Abdominal wall after sutured closure; (**d**) Sutureless closure.

Gastroschisis is one of the most common congenital abdominal wall defects in newborns. Children born with gastroschisis have a full-thickness paraumbilical abdominal wall defect, which is associated with evisceration of bowel and sometimes other organs (Figure 1a). The defect typically measures 2–3 centimeters and is thought to result from involution of the right umbilical vein. Hence, the defect is mostly located to the right of the umbilical cord [1]. The prevalence of gastroschisis has decreased to baseline from 2009 through 2018 after increasing from 2–3 per 10,000 in 1995 to 4–5 per 10,000 in 2005 [2,3,4,5,6,7]. Risk factors associated with gastroschisis include maternal smoking, illicit drug use, alcohol consumption, young maternal age, environmental factors, socio-economic status, opioid prescription rates and others [8,9,10]. It seems likely that changes in gastroschisis prevalence result from changes in risk factor prevalence. Gastroschisis is typically accompanied by intestinal non-rotation and may be associated with intestinal atresia and/or perforations. In contrast to omphalocele, gastroschisis is rarely associated with other congenital or chromosomal anomalies [11]. As the midgut is freely floating in the amniotic fluid, the intestine is typically thickened, edematous and foreshortened [12].

### 1.2. Complex Gastroschisis

Complex (as compared to simple) gastroschisis occurs in about 11–28% of cases and is defined by the presence of intestinal complications including atresia, perforation or necrosis. Intestinal atresia is the most common anomaly associated with gastroschisis occurring in about 10–15% of patients [11,12,13,14].

Complex gastroschisis is associated with increased morbidity and mortality lowering survival rate from >90% to 70–80% in developed countries [15]. Length of stay is dramatically prolonged in complex gastroschisis [16].

The abdominal wall defect may shrink during the last trimester (referred to as closing gastroschisis), this can lead to compression of the herniated viscera resulting in intestinal obstruction and/or mesenteric ischemia. In rare cases, a complete closure of the abdominal wall at birth can be observed. This is referred to as closed gastroschisis. As a result, secondary atresia, the presence of only rudimentary herniated viscera or the complete loss of the herniated bowel (referred to as vanishing gastroschisis) may occur [17]. It is hence crucial to detect signs of abdominal wall closure in utero.

### 1.3. Diagnostic Approach and Prenatal Management

Gastroschisis is typically diagnosed by ultrasound during the second trimester anomaly scan, at around 20 weeks gestational age (WGA). In contrast to omphalocele, the herniated bowel floats freely in the amniotic cavity and is not covered by a membranous hernia sac [18]. First trimester diagnosis of gastroschisis is unreliable, as early ultrasound diagnosis can be confounded by the physiologic midgut herniation leading to false positive results [19]. As physiologic midgut herniation is still present until 12 WGA in up to 20% of fetuses, prenatal diagnosis of gastroschisis earlier in pregnancy needs hence to be interpreted cautiously. The prenatal diagnosis of gastroschisis should be differentiated from other ventral abdominal wall defects, as management and treatment differ between entities. Especially in the unusual cases of in utero rupture of the omphalocele sac, distinction between gastroschises and omphalocele may be difficult. Furthermore, liver herniation—a typical sign of omphalocele—has also been described in cases of gastroschisis with large defects [1]. In contrast, elevation of Alpha Fetoprotein (AFP) in maternal serum is more likely to be observed in pregnancies with gastroschisis as compared to omphalocele [1]. After in utero diagnosis of an abdominal wall defect (gastroschisis or omphalocele), the care of the pregnant mother should be transferred to a tertiary care center for further counselling and treatment by an interdisciplinary team encompassing obstetricians, neonatologists and pediatric surgeons [18]. Serial ultrasounds are performed to evaluate different intestinal outcome parameters (including intestinal dilation and bowel wall thickening), the amount of amniotic fluid and growth of the fetus [18]. Dilation of the intra- (not extra-) abdominal bowel, as well as the presence of polihydramnios are typically observed in complex gastroschisis (with intestinal atresia). Gastric dilation may be associated with increased mortality [18]. An increasing dilation of the small bowel could indicate closing gastroschisis, which necessitates premature delivery in order to avoid loss of the herniated viscera (i.e., vanishing gastroschisis) [20,21] with consecutive short-gut syndrome [22].

### 1.4. Timing of Delivery

Optimal timing of delivery in cases of gastroschisis is strongly debated. When opting for late delivery, it has to be taken into consideration that the herniated intestine is exposed to the toxic environment of the amniotic fluid for an even longer period [23,24]. In addition, the time period of possible intrauterine complications (such as for example, closing gastroschisis) is also prolonged. These factors must be weighed against the general sequelae and complications of preterm delivery. The currently limited evidence shows that elective late preterm delivery (i.e., planned delivery during 35–37 WGA) is associated with less infectious complications and faster toleration of enteral nutrition when compared to expectant management (and delivery at term). In contrast, non-elective preterm delivery is associated with a longer time until normal bowel function (when compared to term delivery) [25]. Furthermore, early delivery at term (i.e., just after 37 WGA) was associated with better outcomes when compared to expectant term delivery [26,27]. Taken together, timing of delivery should be based on the following factors: gestational age (lung maturity), ultrasound findings (fetal growth profile, intestinal outcome parameters) and fetal testing results. In accordance with the available evidence, currently most centers opt for planned delivery at 37 WGA [28,29].

### 1.5. Route of Delivery

The optimal mode of delivery for children with gastroschisis has been much debated. At present, evidence is not sufficient to advocate the use of caesarean section over vaginal delivery for infants with gastroschisis [30,31,32]. Nonetheless, preferred mode of delivery varies between centers and countries. Interestingly in Australia, New Zealand and North America rates of vaginal delivery reach 40–60% [30,33] whereas in northern and central Europe caesarean section is still the preferred mode of delivery for > 90% of children [29,34]. This may be explained by differences in health care systems and local availability and reachability of specialist care.

### 1.6. Postnatal Care

The primary goal of immediate postnatal care is to avoid fluid loss (by evaporation) and hypothermia, as well as to prevent infection. An oro-gastric tube should be inserted to decompress the stomach. Intravenous access is needed for IV fluid resuscitation, sedation and antibiotic administration. The herniated viscera are covered with warm saline soaked gauzes and the lower half of the newborn is placed in a plastic bag. Kinking especially of the mesenteric vessels has to be avoided, the optimal position is on the side in a lateral decubitus position. This is especially important if the patient needs to be transported to another facility. If respiratory support is needed, nasal continuous positive airway pressure (CPAP) or high-flow O_2_ should be avoided (in order to prevent filling the intestine with air). It is better to intubate with an endotracheal tube than using non-invasive modalities for the same reasons as mentioned above. Nevertheless, ventilator support should only be applied if necessary [35] (Table 1).

It is important to notify a pediatric surgeon as soon as possible, ideally before the delivery. Evaluation of the intestine should be done early after delivery to plan the necessary surgical steps.

### 1.7. Surgical Treatment

The primary goal in treatment of gastroschisis is to achieve a timely reduction of the herniated viscera, hereby avoiding harm to the viscera, while averting an abdominal compartment syndrome. To minimize fluid loss and further intestinal impairment, reduction of herniated viscera should be performed as soon as possible [35]. Prior to reduction, the herniated bowel is examined in order to identify intestinal atresia, ischemia or perforation of bowel. Nevertheless, if bowel loops show significant inflammation and are adherent to each other, manual separation should be refrained from in order to avoid injuries to the bowel wall (Table 2).

### 1.8. Primary Versus Staged Reduction

Primary reduction will often lead to higher intraabdominal pressure (IAP) compared to staged reduction. In 50–83% of cases a successful primary reduction without excessive increase of intraabdominal pressure can be achieved [36,37]. A staged reduction reduces duration of mechanical ventilation, risk of infection and time to enteral nutrition [38].

### 1.9. Technique of Staged Reduction

In the past, a silo was created using sterile plastic bags and typically sutured to the abdominal wall. Since 1995 a spring-loaded silo has been made commercially available that is commonly used [39,40,41] (Figure 1b). The intestine is placed inside the silo bag and the ring is placed under the fascia. The closed end of the silo bag can be suspended above the patient [39]. Eviscerated organs are reduced by gravity and with additional manual pressure and the silo volume is gradually reduced over a period of typically 5–7 days. The transparent silo allows herniated viscera to be inspected (for blood supply, perforation, etc.). Even if silo reduction is considered a safe and successful procedure, perforations of the duodenal bowel wall due to compression by the spring-loaded silo [42] and torsions of the mesenteric root upon silo placement have been reported [35].

### 1.10. Technique of Primary Reduction

Depending on the method of subsequent abdominal wall closure, primary reduction may be performed bedside on the ward or in the operating room (OR). Primary reduction with subsequent sutureless closure can be performed bedside (similar to the staged reduction) often using a silo bag with warm saline, whereas primary reduction and sutured closure is typically undertaken in the OR [35].

### 1.11. Abdominal Wall Closure

#### 1.11.1. Sutured Closure

Following reduction the fascia edges are closed using absorbable sutures. It is critical to avoid increased intraabdominal pressure (IAP) (>20 mmHg) after closure of the fascia [43]. In cases of only mild increase of IAP temporary use of muscle relaxants and sedation may be used to reduce fascial tension [35]. If tension and/or IAP prevent primary fascia closure, simple skin closure over the defect or inlay-abdominal wall closure using prosthetic material may be performed [44]. The hereby emerging abdominal wall hernia can be closed at a later point in time (at about 3 years of age) [45]. Alternatively, the umbilical stump may be flapped over the abdominal wall defect and sutured to the fascia edge [46]. Furthermore, a central position of the umbilicus is crucial for a good cosmetic outcome [35] (Figure 1c).

#### 1.11.2. Sutureless Closure of the Abdominal Wall

Sutureless closure may be performed bedside avoiding the need for general anesthesia by applying a watertight non-adherent dressing [47,48,49]. In addition, the umbilical stump may be placed (after cleaning it with betadine) over the defect in order to cover the viscera [50]. Duoderm (from ConvaTec, Deeside, UK) hydrocolloid dressings are hereby used by the authors to cover the gastroschisis defect (Figure 1d). After about 4 days the dressing can be exchanged. When the defect is not leaking any fluid, a permeable dressing can be used, such as, for example, Mepilex border lite (from Mölnlycke Health Care AG, Schlieren, Switzerland). Routine secondary fascia closure should not be necessary. Umbilical hernia after sutureless closure is common and more frequent compared to sutured closure [47]. Although up to about 30% of patients [51] will require umbilical revision at a later time point, spontaneous hernia closure can occur and hernia is generally not associated with significant complications [52]. Outcomes including mortality, length of hospitalization and time to full enteral nutrition are similar following sutureless closure compared to sutured closure [53].

### 1.12. Treatment of Complicated Gastroschisis

Treatment of complicated gastroschisis is often demanding and depends on different factors including comorbidities of the patient, time of delivery, proportion of herniated viscera to abdominal domain and so forth. Hence management needs to be adapted to each individual patient. Nevertheless, and even if a general treatment algorithm is lacking, the following points should be considered when treating patients with complicated gastroschisis: (i) intestinal perforation or necrosis should be addressed surgically as soon as possible, (ii) primary resection and anastomosis is often possible, (iii) resections should be as limited as possible to avoid later short-gut syndrome and (iv) ostomies can be avoided in most cases. Principally the above-named reduction and closure types are also possible in complicated gastroschisis. Hence, both, sutured and sutureless closure techniques may be applied. Likewise, primary reduction is possible in complicated gastroschisis in principle. Nevertheless, in most cases (expectant treatment of intestinal ischemia, intestinal resection with anastomosis, closure of one or more perforations, etc.) a staged reduction in a spring-loaded silo is to be preferred, as it allows daily examination of the bowel and prevents intraabdominal hypertension/abdominal compartment syndrome and hence reduced perfusion of the bowel [42].

### 1.13. Gastroschisis and Intestinal Atresia

About 10–15% of patients with gastroschisis present with concurrent intestinal atresia [12,13,14] with the small intestine being more commonly affected than the colon (80% versus 20% of cases) [42]. As mentioned above, intestinal atresia is not always evident at first presentation and can sometimes only be diagnosed in the course of treatment [42]. Generally, three different treatment options exist: (i) If there are no excessive signs of inflammation/no excessive intestinal peel, the atresia may be resected and primary anastomosis is performed before reduction and abdominal wall closure. If there are excessive signs of inflammation, either a stoma is created (ii) or a silo is fashioned leaving the atresia untouched and resection with anastomosis or stoma creation is performed when abdominal wall closure is performed (i.e., after about 7–10 days) (iii) [13,14]. Whether primary resection and anastomosis and subsequent (primary or staged) reduction should be performed depends on the same basic factors as for all intestinal anastomoses (stable patient, adequate perfusion, freedom from tension at the anastomotic site, absence of distal obstruction, absence of mesenteric twisting and no mismatch of bowel lumens) [54,55].

### 1.14. Postoperative Course

Ileus after reduction is very common and delayed return to oral/enteral nutrition up to 4 weeks is expected and should not lead to unnecessary contrast studies or other procedures/therapies. In simple gastroschisis median time to tolerance of enteral feedings is about 3 weeks [56] and can be significantly longer in complex gastroschisis [57]. If full enteral feeding is not achieved after 4–6 weeks due to persistent vomiting, a contrast study should be performed. If there is evidence of obstruction (e.g., missed atresia) surgical exploration is warranted. In parallel, parenteral fluid-, electrolyte and nutrient substitution should be provided from birth onwards [58].

### 1.15. Complications

In both types of abdominal wall defects (gastroschisis and omphalocele) closure of the defect may lead to abdominal compartment syndrome, which is defined by persistent IAP above 20 mmHg (indirectly measured by bladder pressure) together with loss of function of one or more organs (e.g., anuria) [59]. Particularly in cases of much tension on the fascia closure and/or high ventilation pressure during closure, the treating health care providers should be alerted to a potentially evolving abdominal compartment syndrome. The incidence of necrotizing enterocolitis (NEC) following gastroschisis has decreased to about 5% nowadays compared to earlier studies reporting incidences of about 15% [60,61,62]. Nevertheless, NEC accompanying gastroschisis is mostly mild and does rarely warrant surgical intervention. Despite intestinal non-rotation, incidence of midgut volvulus in gastroschisis patients is fairly uncommon (about 1.2% of cases), likely due to adhesions formed in the course of the disease. More commonly, patients with gastroschisis present with bowel obstruction due to adhesions (20–25%) [51,63], typically in the first year of life but obstruction may also occur later in life [35,64]. Finally, intestinal resections and/or bowel loss due to vanishing gastroschisis may result in short-gut syndrome.

## 2. Omphalocele

### 2.1. Introduction

In contrast to gastroschisis in omphaloceles the abdominal defect is covered by a membranous sac, consisting of three layers: peritoneum, Wharton’s jelly and amnion as the outermost layer. The umbilical cord/vessels insert at the apex of the sac, which typically contains herniated abdominal contents. These can vary depending on the size of the abdominal defect and include intestine, liver, spleen, bladder and/or gonads. The sac covers and protects the herniated organs against harmful external influences. Omphalocele has to be differentiated from gastroschisis, as there are a number of clinical differences between the two entities. Most relevant in terms of prognosis is the difference in associated anomalies. While gastroschisis is rarely associated with other congenital anomalies, patients with omphalocele often have associated congenital or chromosomal anomalies. This in turn has a large influence on morbidity and mortality [65]. Nonetheless, survival in infants with omphalocele has significantly improved over the last years [66].

### 2.2. Embryology and Pathogenesis

The pathogenesis of omphaloceles has not been established so far but there is general agreement on the fact that it must be related to an incomplete closure of the ventral abdominal wall before the 9th week of gestation. Two mechanisms have been proposed to explain the pathogenesis of omphalocele. One theory which is widely accepted is that the extraembryonic gut fails to undergo the obligatory 270° counter-clockwise rotation back into the abdomen during 8–12. WGA, resulting in a small, midline defect [67,68]. Because this theory cannot sufficiently explain cases of larger omphaloceles with herniation of other organs such liver and spleen, a second theory has been put forward. In this, failure of the left and right lateral abdominal folds to close normally as early as in the 3–4. WGA is thought to create a larger abdominal wall defect, through which in turn larger parts of the abdominal cavity can herniate [69,70].

### 2.3. Prevalence and Epidemiology

In contrast to gastroschisis, where an increase in prevalence has been observed over the past years, prevalence of omphalocele has shown to be relatively stable at around 1 in 4000 to 1 in 10,000 live births [6,71,72,73]. Nonetheless, the true incidence of omphalocele has to be estimated higher when fetal demises and terminations of pregnancies are taken into account [73,74,75,76].

Possible maternal risk factors associated with development of omphalocele consist of maternal age < 20 or >35 years [66,77], Afro-American ethnicity [78,79], maternal obesity (BMI > 30 kg/m^2^) [80,81], maternal disorders of glycemic control and in turn fetal macrosomia (>4000 g birth weight) [82] and multiple births [66]. Patient risk factors associated with omphalocele are primarily chromosomal anomalies [83]. Omphalocele is especially common in patients with trisomy 18 (80–90% of cases) [83,84] and Beckwith-Wiedemann syndrome (10–66% of cases) [85,86].

### 2.4. Giant Omphalocele

**Figure 2:**
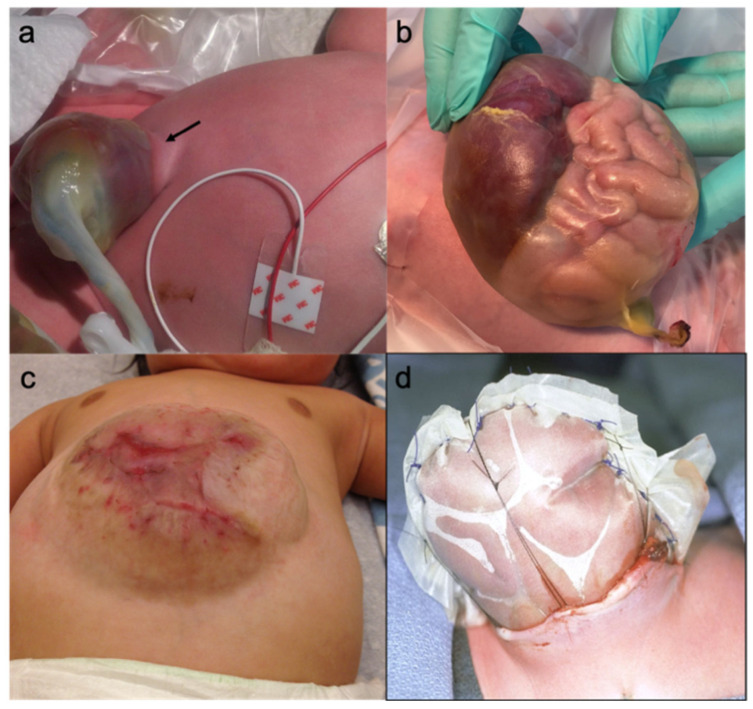
(**a**) Hernia into the cord (arrow pointing at the skin cuff); (**b**) Giant omphalocele with herniated liver and intestine; (**c**) Large abdominal wall hernia after paint and wait; (**d**) Schuster plastic.

Although consistent definitions of large or giant omphaloceles are lacking, they most commonly encompass significant viscero-abdominal size-mismatch. This in turn is associated with higher rates of morbidity and mortality. Generally accepted definition of giant omphalocele is a defect diameter of ≥5 cm and/or herniated liver of >50–75% [87] (Figure 2b). As infants born with giant omphalocele sometimes suffer from pulmonary hypoplasia, this has to be taken into account in postnatal management and therapy [88,89]. Consequently, (anticipated) pulmonary complications should limit early and targeted surgical treatment in these cases.

### 2.5. Hernia into the Cord

The entity of hernia into the cord needs to be clearly differentiated from patients presenting with a small omphalocele as it is rarely associated with chromosomal anomalies and has an excellent prognosis. Typically, children with hernia into the cord present with an intact abdominal wall. Distinctively a cuff of skin is seen extending from abdominal wall onto the neck of the sac (Figure 2a). As opposed to minor omphaloceles where the umbilical ring defect involves not only the skin but also the abdominal wall muscles [90] (Figure 2b). Hernia into the cord is thought to arise from persistence of physiological herniation of the mid-gut beyond 10–12 WGA. At this point development of the abdominal wall has been completed [91]. In both, minor omphaloceles and in hernias into the cord primary surgical treatment and closure of the defect in the first days of life is often possible [92]. Simple umbilical hernias can easily be differentiated from small omphaloceles and from hernias into the cord as they are completely covered by normal skin. As a majority of hernias will close spontaneously within 2 years and their complication (i.e., incarceration) rate is low, repair of umbilical hernias in infants is usually postponed until 4–5 years of age [91,93,94].

### 2.6. Prenatal Diagnosis

Routine prenatal screening and diagnosis of omphalocele and of associated anomalies is considered standard of care and nowadays over 90% of cases are diagnosed prenatally in developed countries [95]. In cases of liver herniation diagnosis of omphalocele with prenatal ultrasound can be made as early as 9–10 WGA. Prenatal diagnosis in cases without liver herniation can be made reliably after 12 WGA [68,96,97,98]. In contrast to gastroschisis, omphalocele is often associated with congenital structural or chromosomal anomalies. Interestingly it is more common to find associated anomalies in cases of small abdominal defects as opposed to giant omphaloceles [99,100].

Once omphalocele has been diagnosed on prenatal ultrasound it is important to look for associated anomalies as a majority of newborns with omphalocele (>70%) will have at least one additional congenital anomaly [34,65,101]. Associated anomalies include cardiac (32%), chromosomal (17%) and central nervous system (8%) defects [102]. Genitourinary anomalies and diaphragmatic hernias are less commonly associated with omphalocele [66]. In order to identify chromosomal anomalies, karyotyping via chorionic villous sampling or amniocentesis can be performed as early as during the first trimester (concomitant risk of early abortion: 0.2–0.3% [103]). Furthermore, prenatal cell-free DNA screening offers a non-invasive method to screen for certain chromosomal abnormalities in a fetus [104,105]. Prenatal fetal organ screening and echography are important tools to identify cardiac and other associated anomalies. If a major chromosomal abnormality is diagnosed and/or other anomalies are present, prenatal parental counselling in a tertiary center is crucial. After birth a close clinical inspection and (repeated) echography are recommended as further anomalies may be identified [66].

### 2.7. Monitoring of Fetal Grwoth

It has been shown that the presence of anomalies in fetuses with omphalocele is strongly associated with poor prognosis and complications leading to fetal demise and neonatal death [34,88,106,107,108]. Fetal growth and wellbeing should be monitored in a tertiary center with serial sonographic controls every 4 weeks (depending on ultrasound findings) up to 32 WGA once diagnosis of omphalocele has been established, as growth restriction is commonly observed and may be an early warning sign of fetal demise [109,110,111]. As fetuses with omphalocele have an abnormally shaped abdominal cavity, classical weight formulae using abdominal circumference may underestimate actual birth weight. This is why for children with abdominal wall defects specific formulae have been developed, encompassing biparietal diameter, occipitofrontal diameter and femur length [112]. To detect signs of late fetal demise serial weekly scans are recommended from 32 WGA until delivery [113,114].

### 2.8. Delivery Timing and Route

Once the decision has been made by the family to continue the pregnancy, a multidisciplinary team involving obstetrics, pediatric surgeons and neonatologists should determine the best timing and route of delivery together with the parents. Against the background of the morbidity associated with prematurity there is no recommendation for preterm delivery as long as fetal wellbeing is not impaired [115]. Optimal route of delivery is still controversially discussed [116,117]. Factors to consider when determining route of delivery are the defect size, organs exteriorized in the sac, the integrity of the sac and any other associated abnormalities. In cases of minor omphalocele vaginal delivery can be considered to be safe. In cases of larger defects or in cases where large parts of the liver are herniated, caesarean section is generally the preferred route of delivery to minimize the risk of significant bleeding, dystocia or sac disruption [118,119]. Small tears in the sac membrane may be primarily closed with a suture or tissue glue if the sac is robust and sufficient to cover the viscera [120].

### 2.9. Postnatal Resuscitation and Care

The omphalocele should be stabilized during resuscitation and transport to avoid bleeding from the liver or congestion of the liver veins. The covering membranous sac should be protected from mechanical injury or desiccation. This may involve covering the sac with sterile saline-soaked gauzes or placing the whole lower half of the infant’s body including the omphalocele into a sterile plastic sac. During primary stabilization care must be taken to avoid hypothermia. A nasogastric tube may be placed or rectal irrigation performed to attain a reduction of the gastrointestinal volume. Vascular access should be established, avoiding the umbilical vessels, with the goal to achieve and maintain euvolemia. Mask ventilation should be avoided and in cases of pulmonary restriction early intubation may be indicated [118] (Table 1).

### 2.10. Postpartal Management

After initial stabilization, an echography should be obtained looking for structural cardiac anomalies and signs of pulmonary hypertension. Furthermore genitourinary, gastrointestinal and anomalies of the nervous system should be evaluated by clinical examination and sonography. If morphological abnormalities are evident on clinical exam, a genetic consultation should be obtained, especially if karyotyping has not been carried out before birth. When Beckwith-Wiedemann syndrome is suspected (macroglossia, macrosomia, hemihypertrophy, history of polyhydramnios or placentomegaly, renal anomalies and/or hypoglycemia) close glucose monitoring should be established [118].

### 2.11. Treatment

The choice and timing of treatment is dependent on the cardiopulmonary state of the infant, associated anomalies, the defect size and the severity of viscero-abdominal disproportion. Goal of any surgical treatment is to cover the fascial and skin defect and avoiding an intolerable increase of intraabdominal pressure. In general, treatment strategies can be classified as following: immediate (primary) repair; staged repair with delayed primary closure; and delayed repair (paint and wait) with secondary closure of abdominal wall hernia (Table 3).

### 2.12. Primary Repair

Hernias into the cord, the majority of cases with small omphalocele and selected cases of larger abdominal defects can be closed primarily during the first days of life. Whenever in doubt, visceral reduction by careful manipulation of the sac contents at the bedside may be helpful to assess tolerance. Primary repair is best carried out in the operating room under general anesthesia. Determination of blood group and single shot antibiotic prophylaxis are done preoperatively. The hernia sac is typically resected and viscera reduced. Of note: upon resection of the hernia sac, conversion into a paint and wait procedure will not be possible anymore. Utmost care must be taken not to cause injury to the liver surface as this may lead to significant bleeding. If the sac is adherent to the liver surface the peritoneal layer of the sac may be left in place to reduce the risk of liver injury. The umbilical vessels are ligated and the fascial defect is closed (vertically, transversely or via purse-string). For the reconstruction of the skinny part of the umbilicus a number of techniques have been described [118,121,122]. Whenever signs of an intolerable increase in abdominal pressure occur (high tension forces on fascial borders and/or persisting increase in ventilation pressures) a change of strategy to a staged repair of the abdominal wall should be considered (see below). In infants with pronounced pulmonary hypoplasia primary repair of the abdominal wall should only be approached after careful evaluation and stabilization of the child [123].

### 2.13. Staged Repair

Treatment options in cases of larger omphalocele with herniation of the liver or a defect measuring ≥5 cm consist of staged- versus delayed repair (paint and wait). Factors influencing decision making are (among others) the degree of abdomino-visceral disproportion and pulmonary hypoplasia.

A number of techniques have been described, with the goal to achieve a gradual visceral reduction over the course of 5–10 days, after which the abdominal wall can be closed. If preserved, the amniotic membrane may serve as antiseptic barrier and silo. Furthermore, a change of the treatment strategy to a paint and wait procedure is still possible at any stage. Infants undergoing staged repair will often need mechanical ventilatory support, adequate sedation with or without neuromuscular paralysis and parenteral nutrition until repair of the abdominal wall [118,124]. Procedures should be generally undertaken in the operating room under general anesthesia with optimal monitoring of cardiopulmonary status and intra-abdominal pressure. Especially patients without pulmonary hypertension and/or rupture of the sac membrane qualify for staged reduction [125].

Historically children with large omphaloceles were managed using mobilized skin flaps to cover the exposed viscera. The resulting large ventral hernia was then corrected at a later time [126]. In 1967 Schuster et al. described the use of a Silastic silo to allow for staged reduction. In this technique the omphalocele sac is excised and the silastic sheeting is sewn to the rectus fascia or to the full thickness abdominal wall [123,127] (Figure 2d). Serial reduction is performed by clamping and suturing or application of a linear stapler at the silo apex, until a delayed closure is achieved [128,129]. Intraabdominal tissue expanders may be used to create further abdominal space [130]. Primarily described by deLorimer, visceral reduction can also be achieved by serial wrapping and compression of the omphalocele sac with lipid gauzes and elastic bandages [131]. After successful visceral reduction the sac can be excised and primary closure or mesh closure performed. A tear in the sac can be potentially salvaged by suturing it back together [132]. Fascial closure may further be facilitated by fascial advancement and component separation as described by Ramirez et al. [133,134].

Various materials have been used for fascial closure in cases of large abdominal defects, including biologic and synthetic meshes [115]. Serial excisions of the central portion of the mesh may eventually allow for native fascial closure. If native closure is not possible, a mesh may be interposed to close the abdominal wall defect. Initially mainly nonabsorbable materials such as Teflon, polytetrafluoroethylene, polypropylene and polyester were used [135,136]. The fairly high rate of mesh-related complications (~25%) such as erosions causing chronic inflammation, foreign body reactions and fistulas, as well as infections have led to the development of absorbable and biologic mesh materials, mostly derived from human or porcine acellular dermis with scaffolds of collagen or elastin [123]. These materials have the advantage of growth factors facilitating in growth of tissue and vessels at the implantation site [123,135]. Resorption of the mesh can take weeks to months. Compared to nonabsorbable mesh types, infectious complications are less commonly reported, whereas hernia recurrence, most likely due to the resorption of the mesh, seems to be more common [123]. Although the use of mesh for serial reduction of the abdominal wall defect may be a good alternative to the Silo technique, especially in large defects (where a spring-loaded silo is easily displaced), the use of mesh for definitive closure of the fascia should be well balanced against the background of possible complications. Especially when the defect is small and centrally located it may be best to avoid mesh implantation altogether and accept a small ventral hernia that can be closed later. Thus, in some cases only skin flap closure over the abdominal defect may be warranted [126]. If skin closure over the mesh is not possible, tissue expanders can be placed lateral to the defect in a subcutaneous pocket to create skin flaps [137]. Furthermore, vacuum-assisted closure (VAC) type dressings and antibiotic ointments over biologic meshes have been reported as successful techniques facilitating incorporation and skin growth. Taken together, outcome data are difficult to assess, as only small case series have been published for each technique and little comparative data is available [135].

### 2.14. Delayed Repair (Paint and Wait)

The strategy of delayed repair is often chosen for infants with giant omphalocele and/or a high degree of abdomino-visceral disproportion. Further patients qualifying for this treatment are children with low or very low birth weight, with marked pulmonary hypoplasia and/or other comorbidities [138]. The goal of the paint and wait technique is to achieve an escharization and eventual skin coverage of the viscera. In contrast to primary and staged repair, delayed repair always results in an abdominal hernia (Figure 2c) which is closed in a second step once the child has grown (from 6 months to 3 years of age). To prevent infectious complications and to promote escharization, topical combined escharotic/anti-infective agents can be applied. Silver sulfadiazine preparations and povidone iodine solutions hereby are the most commonly used agents [138,139]. The hernia sac is daily painted with the escharotic agent and covered with dressings to protect the membrane and to avoid rupture. Compared to staged repair infants undergoing paint and wait have a shorter time to full enteral feeds and there is often no need for mechanical ventilatory support or neuromuscular paralysis [140].

### 2.15. Long Term Complications and Outcome

Complications often observed in children after repair of omphalocele are gastroesophageal reflux disease, feeding difficulties, failure to thrive and chronic lung disease. Furthermore, in children with giant omphalocele neurodevelopmental and motor delays occur more often [141]. Apart from abdominal hernias, as a result from increased intraabdominal pressure, children after omphalocele repair also have a higher incidence of inguinal hernias [142]. Although not common, volvulus does occur after repair of omphalocele in up to 3% of cases. Thus, it is important to be aware of this differential diagnosis in children presenting with acute abdominal pain and bilious vomiting after omphalocele repair [143]. More commonly (13–15%) patients present with adhesive small bowel obstructions [144].

Outcome and survival have drastically improved over the last decades in children born with omphalocele. Overall survival for liveborn omphalocele infants is up to 80% [145]. In cases of isolated omphalocele one year survival rate has been reported as high as 90% [66].

## Figures and Tables

**Table 1:** Abdominal wall defects—overview.

	Gastroschisis	Omphalocele
Location	Mostly right of umbilical cord	Midline
Etiology	Involution of right umbilical vein	Failed omphalomesenteric duct involution vs. failed lateral abdominal wall closure
Associated disorders/anomalies	Intestinal Atresia (10–15%), perforation, necrosis	Cardiac (32%), chromosomal (17%), CNS (8%), other (GU, GI, musculoskeletal, eyes, ears, face, neck (21%)). Isolated omphalocele without anomalies (22%)
Special forms	*Complex gastroschisis*: Gastroschisis associated with intestinal disorders (atresia, perforation, etc.), 11–28% *Closing/closed gastroschisis*: abdominal wall defect getting smaller, strangulated eviscerated bowel, towards end of pregnancy *Vanishing gastroschisis*: Involution of the eviscerated bowel (due to closed gastroschisis)	*Giant omphalocele*: abdomino-visceral disproportion, no clear definition (defect ≥ 5 cm and/or herniated liver of >50–75%) *Hernia into the cord*: Cuff of skin at base of sack, normal abdominal wall muscles, rarely associated disorders, primary closure often possible.
Prenatal ultrasound	Free floating herniated bowel, umbilical cord insertion in abdominal wall, seldomly liver herniation	Membranous sac covering viscera (prenatal sac rupture possible), umbilical cord insertion into sac, liver herniation
Mode of delivery	vaginal or caesarean	Small omphaloceles: vaginal. Large omphaloceles/herniated liver: caesarean
Timing of delivery	Elective late preterm or early term (35–38 wGA)	At term
Postnatal resuscitation and care	Place saline soaked gauzes around defect Plastic bag around lower ½ of patient Get IV access (avoid umbilical vein) Avoid hypothermia Oro- (or naso-) gastric (+ rectal) decompression Avoid mask ventilation (no intubation by default)	

wGA = weeks of gestational age, IV = intravenous, CNS = central nervous system, GU = genitourinary, GI = gastrointestinal.

**Table 2:** Gastroschisis treatment.

Reduction of simple gastroschisis	Staged- seems favorable over primary reduction
Closure of simple gastroschisis	Sutured (needs general anesthesia, more infections) versus sutureless (more hernias) closure
Reduction of complicated gastroschisis	Staged reduction (spring-loaded silo)
Closure of complicated gastroschisis	Sutured versus sutureless
Gastroschisis and atresia	Primary resection/anastomosis vs. ostomy formation vs. delayed primary closure (after silo treatment)
Postoperative course	Time to return of bowel function: up to 4 weeks
Complications	Abdominal compartment syndrome, NEC (mostly benign), midgut volvulus (seldom), adhesive Small bowel obstruction (first year of life)

NEC = necrotizing enterocolitis.

**Table 3:** Omphalocele treatment.

Repair of hernias into the cord/small omphaloceles	Primary repair
Repair of larger (giant) omphaloceles without abdomino-visceral disproportion/pulmonary hypertension	Staged repair (e.g., Schuster)
Repair of giant omphaloceles with abdomino-visceral disproportion/pulmonary hypertension	Paint and wait
Complications	Gastroesophageal reflux, feeding difficulties, Failure to thrive, chronic lung disease, inguinal hernias, abdominal compartment syndrome, midgut volvulus (seldom), adhesive small bowel obstruction
